# Practical Remarks on the Diseases Which Occurred on Board of His Majesty's Ship, Astrea, on the Jamaica Station, during the Years 1787, 1788, 1789, and Part of 1790

**Published:** 1799-04

**Authors:** Stewart Henderson

**Affiliations:** formerly a Surgeon in the Royal Navy; now of the Army Hospital Staff


### *Practical Remarks on the Diseases which occurred on board of His Majesty's Ship, Astrea, on the Jamaica Station, during the Years* 1787, 1788, 1789, *and Part of* 1790*: By* Stewart Henderson, *formerly a Surgeon in the Royal Navy; now of the Army Hospital Staff.*

### INTRODUCTION.

HAVING been professionally employed in various situations and countries with large fleets and armies, where there was an extensive field for medical observations, I had an opportunity of witnessing disease in every shape, and had often to lament, that the means of relief were so inadequate, not only from a deficiency of useful medicines, but such auxiliaries as can alone render the practice of physic succesful. However, within these few years, great improvements have taken place in the medical department of the navy, (much to the credit of those who preside over that service) by a gratuitous supply of medicines, and every comfort necessary to preserve and restore the health of that valuable class of men to the state—the seamen of the royal navy. The situation of the surgeons has likewise been improved, and the posts of honour and emolument multiplied, by the physicians to the fleets and hospitals having been *all* taken, this war, from the list of navy-surgeons, which has brought forward men of knowledge and experience, who had before declined serving; and induced men of abilities to come into the service, as their writings, on professional subjects, prove. But what is a better criterion, the succes of their practice in surgery (as well as in physic), which the memorable victories gained by the British fleets, gave them an opportunity of shewing—These circumstances will, no doubt, have due weight with those who have it in their power to improve their situation; so that the public may reap the full extent of their studies and professional exertions, which will ultimately promote the welfare of his majesty's service, and contribute to the preservation of many lives.

But when we look into the medical department of the other ſervice, we find that the ſick of the Britiſh army (with very few exceptions) in every part of the world, particularly in the Weſt-Indies, on the continent, and at the Cape of Good Hope, have been entirely under the care of men, who never held any employment in his majeſty's ſervice, until they were appointed phyſicians during this war. In juſtice, however to thoſe gentlemen, we muſt allow, they came from a learned reſpectable body, and might poſſeſs profound medical erudition; but it has been fatally experienced, that no courſe of lectures, no reading, can qualify a man for medical ſervice in hot climates: for medicine being a conjectural art, can only be learned from extenſive practice, experience, and obſervation, in various climates. It muſt therefore appear obvious to every judicious, impartial, and unprejudiced man, that there was a great impropriety in ſending phyſicians to foreign climates, who had no local knowledge of the countries, or their diſeaſes; and were totally unacquainted with the habits and manners of ſoldiers; a knowledge eſſentially neceſſary to a military phyſician or ſurgeon.

From the great attention ſhewn by his Royal Highneſs the Commander in Chief, to the arrangement and improvement of every department of the army, there is the beſt reaſon to expect, that a grievance no leſs injurious to the public ſervice, than to individuals, will ere long be remedied. And it is to be hoped from his Royal Highneſs's conſideration to whatever is ſuggeſted for the relief of the ſoldier, the period is not diſtant, when the ſick of the Britiſh army will enjoy equal medical advantage with the ſeamen of the royal navy. No good reaſon, I imagine, can be aſſigned why they ſhould not. Policy—humanity, dictates it; and in juſtice to thoſe army ſurgeons, who have been many years in his majeſty's ſervice, and have endeavoured to merit preferment, by profeſſional exertions, a diligent and faithful diſcharge of their duty.

It is evident, from the nature and ſtructure of the human body, and what we know of the laws which govern the animal economy, derangements inducing diſeaſe will ever occur from unavoidable cauſes, in ſpite of every human exertion; though their danger may be obviated, and ſome of them, no doubt, entirely prevented, by means which are certainly in our power.

PNEUMONIA.

The firſt diſeaſe which occurred was pneumonia, or inflamation in the lungs, and ſeemed to be occaſioned by the cold, boiſterous weather we met with in the channel. The patients complained of violent pains in the thorax, cough, and difficult reſpiration; pulſe frequent, and every ſymptom of pyrexia. In the beginning, bleeding was made uſe of, which afforded relief; relief; a purgative was given, and a bliſter applied to the thorax; the ſp. minder. with acet. ſcillit. and ſometimes the vin. antimon. were given, with a view to promote perſpiration and expectoration, which came on about the third or fourth day, and kept up by acidulated and demulcent drinks, ad libitum. In the convaleſcent ſtate, bark and wine, with a nouriſhing diet, were ordered, to recover from the debility, which the evacuations, and copious expectorations had occaſioned. Our coming into the temperate lattitudes ſeemed to accelerate the removal of theſe complaints; except in one man, a marine, who, after we left Madeira, informed me, that he was very much troubled with a cough, and ſpitting of matter. Upon examination, it had every appearance of proceeding from an ulcer; his pulſe quick and frequent, and profuſe ſweats in the night; he acknowledged to have been ill a twelve-month, and had only returned from the hoſpital a few days before he embarked; he being told by the ſurgeon, whoſe care he was under, that he might recover, if he went to a warm climate, which was his motive for not making his illneſs known when he came on board, for fear he would have been returned to quarters. Had he applied to me before we left Madeira, I would have requeſted to have had him left on that iſland, where he would have had every chance of recovery, from the mild temperature of the climate; as his diſorder was far advanced, and the heat of the weather increaſing the deblity, I had little hopes of his living to reach Jamaica. To mitigate particular ſymptoms, ſuch as cough, pain, and ſtricture in the thorax, an antimonial anodyne draught was given every night, a bliſter was applied to the pained part, and kept open, with a view to heal the ulcers in the lungs. I attempted it on the principle of ſupporting and reſtoring the general health; the Peruvian bark was given, with ſome preparation of ſteel; rice and ſago for diet, with ſometimes a bit of fowl, and good Madeira wine, humanely allowed from Capt. Rainier's table, and often from the officers' meſs.

On our arrival at Port Royal, he appeared to have gained ſtrength; but, from being much diſturbed by the noiſe on board, I thought it beſt to ſend him to the hoſpital, that he might have the benefit of a milk-diet, reſt, and quietneſs; but he died ſoon after. Upon the whole, from this man's caſe, and many others, which have ſince fallen under my obſervation, I cannot recommend a hot climate for confirmed pulmonary conſumption.

### RHEUMATISM.

This diſeaſe, to which ſeamen are particularly ſubject in northern latitudes, when expoſed, after being over heated, to the ſudden influence of cold, is diſtinguiſhed into the acute or chronic: the latter is often feigned, and not eaſily diſcovered by the moſt diſcerning phyſician. This complaint was was the confequence of the cold moift weather, which produced pneumonia. The firft patient was of a plethoric and rigid fibre, complained of univerfal pains, efpecially in the joints, which were moft fevere when warm in bed; pulfe frequent and hard; thirft increafed. Bleeding was freely made ufe of, which relieved the pain, and abated the febrile fymptoms; a purgative was given, and he took one fcruple of the pulv. dover. every hour, until a plentiful perfpiration came on; it was kept up, and encouraged by drinking balm or fage-tea; by repeating the fudorific, his fever and pains left him, except in one of his knees, which a blifter removed.

The other cafe which occurred was of a chronic nature. The patient chiefly complained of pains affecting the lower joints, without febrile fymptoms; he was of a relaxed and debilitated habit. Upon inquiring into his character, I had no reafon to fuppofe that his complaint was not real. I put him on a better diet, with wine, and to take 40 drops of the tinct. guiac. volat. with 20 drops of tinct. opii, twice a day; at the fame time, to ftrengthen the fyftem, and reftore the parts to their proper tone, bark and fteel were given, and warm ftimulating applications to the pained parts. By a continuance of thofe ftimulants and tonics, all his fymptoms were removed; perhaps affifted by the warm weather, for he had no return of them, during the reft of the ftation; a proof that a hot climate is favourable for preventing the attack and recurrence of this difeafe. Had his complaints not given way to thefe remedies, I intended to have given him calomel, as an alterative, having found it of great fervice in feveral cafes of rheumatifm, efpecially when there was reafon to fufpect a venereal taint in the habit.

REMITTENT, OR MARSH FEVER.

This fever, the legitimate offspring of all hot climates, efpecially where marfhes abound, is the autumnal difeafe of moft parts of Europe, only appearing in a milder degree. It has been defcribed by authors under various names, *bilious*, *yellow*, *Jamaica*, *Senegal*, and in Bengal, *pucka;* but multiplying diftinctions which do not exift, only ferves to perplex and miflead; for it will be found to be the fame individual difeafe, under different modifications, depending on conftitution, feafon of the year, and local fituation. The caufe of this fever, in all its varieties, is marfh effluvia; nor can, in my opinion, any other caufe produce it. We find that, in fome places at the Cape of Good Hope, where no fuch caufe exifts, this fever is unknown. We likewife find, that ftrangers are more liable to be affected by this noxious effluvia, and have the difeafe in a more formidable degree than the natives of the country, whofe conftitutions acquire a certain power of refifting it from habitual expofure; at the fame time, its effects on them are obvious, by fhortening

ſhortening the duration of life. I do not think that the original diſeaſe produced by this miaſma is infectious, but that it may alter its type, and become highly contagious from concurrent cauſes; as from too many diſeaſed bodies being crouded together, without paying ſufficient attention to ventilation and cleanlineſs. This appears to have been the caſe in the Britiſh hoſpitals, in every part of the world, and occaſioned the great mortality which has prevailed in the army, particularly in the Weſt Indies, and on the Continent. When a man was ſent into theſe hoſpitals, however ſlight his complaint, it degenerated, in a very few days, into the moſt malignant fever; and, as Dr. SINNOT very juſtly remarks, there was leſs danger in entering into the field of battle, than attending our ſick in the general hoſpitals. This noxious exhalation enters the ſyſtem, either by the lungs, ſkin, or ſtomach; but the manner in which it produces thoſe ſymptoms of diſeaſe, which characterize the fever, does not appear to be well underſtood. We can only perceive its general effects on the ſyſtem; and that it may lurk for a certain time in the habit, before morbid movements take place. Several inſtances of this occurred when I was laſt at Jamaica. His Majeſty's ſloop of war, Alert went up to Rock Fort to water, and anchored cloſe to the ſhore, when the atmoſphere was charged with marſhy exhalations; her crew were then in perfect health. She immediately went to ſea, and fourteen days after, the greateſt part of the men were attacked with the remittent fever, and it was obſerved, that the men who had moſt acceſs to the ſhore, were the firſt ſeized with the complaint. The ſubjects on whom this fever exerts its greateſt violence, are of two different temperaments; firſt, the young and plethoric, and thoſe of a rigid, irritable fibre, and great excitability, have every thing to fear from expoſure to the action of this poiſon, eſpecially when the body has been overheated, either by the uſe of ſtimulating liquors, or by violent exerciſe in the ſun; ſecondly, thoſe who are under the impreſſion of fear or diſappointment, and are conſtitutionally deficient in energy of mind and body, who may be ſaid to be conſiderably under the ſtandard of health, and eaſily affected by every debilitating power.

As this exhalation is found to exert its influence to a conſiderable diſtance, notwithſtanding every precaution to prevent it, thirty of our men felt its effects on board of ſhip. The patients of the firſt claſs were young men (chiefly marines) of athletic habit, who had never been in a hot climate. They complained of nauſea, ſickneſs, violent pain in the head, particularly in the fore-part, and ſtrong pulſation of the temporal and carotid arteries; the eyes became red and turgid, with evident determination of the blood to the brain. The lungs and ſtomach appeared to be in a ſtate of inflammation, and great irritability: the latter rejecting every thing taken into it; the tongue white; white; ſkin dry; the pulſe quick and full. From the violence and rapidity of the ſymptoms affecting organs of primary importance, I conſidered it neceſſary that ſomething deciſive ought immediately to be done, before the morbid movements had made too great progreſs. With a view to relieve inflammatory ſymptoms, and prevent the brain being oppreſſed, bleeding was freely reſorted to, and not by any determined quantity, but until the ſymptoms ſubſided, which was manifeſt from the pulſe becoming ſoft and regular. Cool air was freely admitted, and cold applications to the head were directed to be uſed; after bleeding, a large bliſter was applied to the region of the ſtomach, which removed the pain and irritation of that organ. Calomel, with James's powder were adminiſtered to procure ſtools, and promote perſpiration; this was encouraged by drinking, *ad libitum,* of balm or ſage tea. In the convaleſcent ſtate, bark, wine, and a nouriſhing diet, were had recourſe to, which removed the debility brought on by the violence of the ſymptoms, as well as that occaſioned by the neceſſary evacuations.

The ſecond claſs were men of a lax fibre, and diminiſhed energy of mind and body, with a conſiderable degree of nervous irritability, and but little vaſcular excitement. The effects of the virus upon theſe, were giddineſs, ſickneſs, and diſagreeable ſenſation at the ſtomach, attended with frequent vomiting of a ropy matter; the head much affected; the pulſe quick, irregular, ſometimes tenſe; the countenance deſponding; the ſkin dry; the tongue white; the belly torpid; the ſecretion of bile diminiſhed; the urine ſcanty and limpid. As there was no inflammatatory diſpoſition inducing dangerous determinations of the blood, bleeding was not employed; although there was nauſea and ſickneſs. Antimonial emetics were not uſed, having always obſerved that they increaſed the irritability of the ſtomach, which is the moſt troubleſome ſymptom attending this form of the fever; a bliſter ſometimes had the beſt effect, when applied to the region of the ſtomach, and, in ſome caſes, over the whole of the head. Camomile tea, and the efferveſcing draughts were given, with a view to relieve the nauſea, and vomiting: to promote perſpiration, James's powder, with camphor, were adminiſtered every hour, until the effect was produced. The bowels were opened by rhubarb and calomel; to ſupport and increaſe the energy of the ſyſtem, bark and wine were adminiſtered, as ſoon as the ſtomach had the power of retaining them, and continued with a nouriſhing diet, until health was perfectly reſtored.

There is another form in which this poiſon acts on ſubjects partaking neither of the violent inflammatory ſymptoms of the firſt, nor the diminiſhed energy or nervous affection of the ſecond. Men, who may be ſaid to be neither above nor below the common ſtandard of health, are able ſometimes to to overcome the influence of this noxious vapour; but, when the habit was not vigorous enough to refift it, they were generally feized with cold fhivering, fucceeded by great heat, ficknefs, and fometimes a vomiting of bile; the tongue white and furred; the pulfe quick and full; thirft great; urine high-coloured; although there were marks of irritation and inflammatory diathefis in the beginning, but yet not fufficient to juftify blood-letting; which, I confidered, would have diminifhed the vital power, and reduced the patient to the fame ftate as defcribed in the fecond form. I therefore thought it more advifeable to employ mercurial purgatives, which had a very good effect in carrying off the bilious fordes collected in the firft paffages, and fupporting, in a great degree, the febrile phenomena;—Emetics were fometimes given; James's powder, with camphor, to promote perfpiration, and effect a complete remiffion. After the bowels were emptied, and the fudorific procefs completed, the bark was immediately given, and continued day and night, which generally checked the progrefs of the difeafe. Wine, and a nourifhing diet, in the convalefcent ftate, were always allowed. In concluding thefe few remarks on the varieties of the endemic fever, I muft obferve, that the firft form of the difeafe appears only in fubjects newly arrived from Europe, the young and plethoric; for a refidence in a hot climate, and other debilitating caufes, remove that rigidity of fibre, and denfity of the fluids, which co-operated at firft in producing thofe violent derangements. Men of this defcription fhould therefore, before their arrival, undergo fome preparation to reduce their habits; while thofe of the fecond clafs, ought to refort to means which may give a certain degree of ftrength and energy to the fyftem. They will then have the difeafe in a milder degree, fuch as it appears in thofe fubjects attacked with the third form of the diforder.

(To be continued in our next Number.)

